# RNAi-Mediated Silencing of Atp6i and Atp6i Haploinsufficiency Prevents Both Bone Loss and Inflammation in a Mouse Model of Periodontal Disease

**DOI:** 10.1371/journal.pone.0058599

**Published:** 2013-04-05

**Authors:** Hongbing Jiang, Wei Chen, Guochun Zhu, Lijie Zhang, Byron Tucker, Liang Hao, Shengmei Feng, Hongliang Ci, Junqing Ma, Lin Wang, Philip Stashenko, Yi-Ping Li

**Affiliations:** 1 Department of Pathology, University of Alabama at Birmingham, Alabama, United States of America; 2 College of Stomatology, Nanjing Medical University, Nanjing, Jiangsu Province, People's Republic of China; 3 Harvard School of Dental Medicine Department of Restorative Dentistry and in Endodontics, Boston, Massachusetts, United States of America; 4 Department of Immunology and Infectious Disease, The Forsyth Institute, Cambridge, Massachusetts, United States of America; 5 Department of Clinical Laboratory, The Third Hospital of Hebei Medical University, Shijiazhuang, Hebei Province, People's Republic of China; University of Toronto, Canada

## Abstract

Periodontal disease affects about 80% of adults in America, and is characterized by oral bacterial infection-induced gingival inflammation, oral bone resorption, and tooth loss. Periodontitis is also associated with other diseases such as rheumatoid arthritis, diabetes, and heart disease. Although many efforts have been made to develop effective therapies for this disease, none have been very effective and there is still an urgent need for better treatments and preventative strategies. Herein we explored for the first time the possibility that adeno-associated virus (AAV)-mediated RNAi knockdown could be used to treat periodontal disease with improved efficacy. For this purpose, we used AAV-mediated RNAi knockdown of Atp6i/TIRC7 gene expression to target bone resorption and gingival inflammation simultaneously. Mice were infected with the oral pathogen *Porphyromonas gingivalis* W50 (*P. gingivalis*) in the maxillary periodontium to induce periodontitis. We found that Atp6i depletion impaired extracellular acidification and osteoclast-mediated bone resorption. Furthermore, local injection of AAV-shRNA-Atp6i/TIRC7 into the periodontal tissues *in vivo* protected mice from *P. gingivalis* infection-stimulated bone resorption by >85% and decreased the T-cell number in periodontal tissues. Notably, AAV-mediated Atp6i/TIRC7 knockdown also reduced the expression of osteoclast marker genes and inflammation-induced cytokine genes. Atp6i^+/−^ mice with haploinsufficiency were similarly protected from *P. gingivalis* infection-stimulated bone loss and gingival inflammation. This suggests that AAV-shRNA-Atp6i/TIRC7 therapeutic treatment may significantly improve the health of millions who suffer from *P. gingivalis*-mediated periodontal disease.

## Introduction

Periodontitis is a common chronic inflammatory disease that is induced by polymicrobial infection, with a prominent pathogen being *Porphyromonas gingivalis*. The pathogenesis of periodontal disease involves bacterial biofilms that develop on the tooth surface and within the gingival crevice, which induces a host inflammatory response in the gingival tissues. The response results in osteoclast-mediated bone loss and the consequent loss of teeth. In addition, periodontitis is associated with numerous systemic diseases, including stroke, diabetes, heart disease, and adverse pregnancy outcomes. An investigation conducted by Kawai *et al.* that activated T- and B-cells in the gingival tissues are the primary sources of receptor activator of nuclear factor kappa-B ligand (RANKL) that induce osteoclastogenesis, osteoclast activation, and bone loss in periodontitis [1]. Inhibiting the function of RANKL produced by activated T-cells can prevent alveolar bone loss [2]. These studies provide strong evidence that T-cell activation mediates bone loss via recruitment and activation of osteoclasts. Osteoclasts remove bone by extracellular acidification of the mineralized bone. Osteoclasts are able to decrease the pH at the interface with bone via a multi-unit vacuolar-type H^+^-ATPase (V-ATPase) complex, which is necessary for osteoclast mediated bone resorption [3], [4]. An isoform of Atp6i, T-cell immune response cDNA7 (TIRC7), is expressed specifically in T-cells as a transmembrane protein that is up regulated during T-cell activation [5], [6]. Functional studies of TIRC7 *in vitro* and *in vivo* via TIRC7^−/−^ null mice have indicated that TIRC7 has a significant association with the regulation of T- and B-cell activation [7]. In addition, several studies have demonstrated increased survival of organ allograft transplants with anti-TIRC7 mAb therapy [8], [9].

The gene transcript for Atp6i and TIRC7 is located on chromosome 11q13, and it is alternatively spliced depending on which cell type (*i.e.* T-cell or osteoclast) the gene is transcribed and expressed in. There are 518 unique base pairs in exons for the TIRC7 transcript in T- and B-cells, and there are 690 base pairs unique to the Atp6i transcript in osteoclasts [6]. However, there are 1,939 base pairs shared by the Atp6i and TIRC7 transcripts in T- and B-cells and osteoclasts [6], which provide shared sequences that can be used for viral vector mediated-RNAi knockdown for dual silencing of Atp6i in osteoclasts and TIRC7 in T-cells.

The viral vector of choice in current translational investigations has been the adeno-associated virus (AAV), which has been proven successful in humans [10]. This approach has been proven safe and well tolerated by patients with advanced Parkinson's disease and only causes a very mild immune response [11]. Furthermore, recent studies have demonstrated AAV's impressive ability to be effective long-term in varying doses [12], including local tissue knockdown, allowing for gene therapy *in vivo* [13]. Therefore, we used AAV-mediated RNAi to investigate the effect of simultaneous Atp6i/TIRC7 silencing.

## Materials and Methods

### Ethics statement

All experimental protocols were approved by the NIH and the Institutional Animal Care and Use Committee of UAB and completed within 16 weeks after birth of mice [14], [15]. Approval for the animal protocol related to this study (Animal Protocol Number 11090909236) was renewed by the University of Alabama at Birmingham (UAB) Institutional Animal Care and Use Committee (IACUC) on August 15, 2011.

### Cells and cell culture

Pre-osteoclasts and mature osteoclasts in primary culture were generated from mouse bone marrow (MBM) as previously described [4], [16], [17]. Briefly, MBM was obtained from tibiae and femora from six-week-old female WT BALB/cJ mice and Atp6i^+/−^ mice [18], [19]. MBM cells (1–2×10^5^) were seeded into wells of a 24-well plate and 1×10^6^ MBM seeded into wells of 6-well plate. MBM was cultured in *á*-modified Eagle's medium (α-MEM; GIBCO-BRL) with 10% fetal bovine serum (FBS; GIBCO-BRL) containing 20 ng/ml macrophage colony-stimulating factor (M-CSF) (R&D Systems). After 1 day, cells were further cultured in the presence of 10 ng/ml RANKL (R&D Systems) and 10 ng/ml M-CSF for an additional 4 days to generate mature osteoclasts. If cells were cultured on bone slices, then an additional 2 days were needed.

### Design and construction of short hairpin ribonucleic acid (shRNA)

Using the Dharmacon siDESIGN Centre (http://www.dharmacon.com) [4], we generated shRNA that would simultaneously target exon 15 of Atp6i and exon 10 of TIRC7. As a control vector, we used AAV-H1-shRNA-luc-YFP (gift from Dr. Sonoko Ogawa), which contains a luciferase-specific shRNA and a yellow fluorescent protein (YFP) cassette [20]. AAV-H1 contains a human Pol III H1 promoter for expression of shRNA as well as an independent green fluorescent protein (EGFP) expression cassette [13]. We cloned the H1 promoter shRNA expression cassette into the AAV construct as described [13], [21], [22]. The following shRNA oligonucleotides were annealed and cloned downstream of the H1 promoter of AAV-H1 into BglII and HindIII sites to produce AAV-H1-shRNA-Atp6i/TIRC7:5′GATCCCCGTATCCTCATTCACTTCA **TTTCAAGAGA** ATGAAGTGAATGAGGATACTTTTTGGAAA-3′ Nucleotides specific for targeting Atp6i/TIRC7 are underlined. The bold type signifies the 9-base pair hairpin spacer.

### AAV RNAi viral production and purification

We used the AAV pHelper-Free System (AAV Helper-Free System, Stratagene) for viral production, which was accomplished using a triple-transfection, helper-free method, and purified with a modified version of a published protocol [21]. Briefly, HEK 293 cells were cultured in 150×25 mm cell culture dishes and transfected with pAAV-shRNA, pHelper and pAAV-RC plasmids (Stratagene) using a standard calcium phosphate method. Cells were collected after 60–72 hours and lysed via shaking with chloroform at 37°C for 1 hour. Sodium chloride was then added and shaken at room temperature for 30 minutes. The stock was spun at 12,000 RPM for 15 minutes and the supernatant was collected and cooled on ice for 1 hour with PEG8000. The solution was spun at 11,000 RPM for 15 minutes, and the pellet was treated with DNase and RNase. After the addition of chloroform and a five minute centrifugation at 12,000 RPM, the purified virus was in the aqueous phase at viral particle numbers of approximately 1×10^10^/ml. The AAV particle titer was determined using the AAV Quantitation Titer Kit (Cell Biolabs, Inc.). To confirm the effect of silencing, we examined the expression of Atp6i in osteoclasts using qRT-PCR, Western blot, and immunofluorescence techniques. Luciferase expression vector AAV-Luc was purchased from North Carolina University. The AAV-luc was injected into the right side of the lower jaw and 14 or 35 days later luciferase expression was measured by an IVIS Imaging System 100 Series (Xenogen Corporation, Alameda, CA) as previously described by us [23].

### Animals

Eight-week-old female wild-type (WT) BALB/cJ mice (Jackson Laboratory) were used for this study. As detailed in Table S1 in File S1, mice were divided into 4 groups: (1) Normal group (no *P. gingivalis* infection) (n = 7 mice); (2) *P. gingivalis* W50 infection and PBS treatment (disease control) (n = 7); (3) *P. gingivalis* infection and AAV-shRNA-Atp6i/TIRC7 (hereafter referred to as AAV-sh-Atp6i) treatment (n = 7); (4) *P. gingivalis* infection and AAV-sh-luc-YFP treatment (negative control) (n = 7). The experiments were performed in triplicate on three independent occasions, resulting in a total sample number of N = 21 for each group. Heterozygous Atp6i^+/−^ mice previously generated by our lab [3], were backcrossed for 4 generations from the C57BL/6J to the BALB/cJ genetic background. The estimated genetic heterogeneity is about 93.75% for mice with a pure BalB-C background. After four generations of backcrosses, the purity of the background is sufficient and comparable to wild-type mice. Thus, the background would not affect the experimental findings. As detailed in Table S2 in File S1, eight-week-old heterozygous Atp6i^+/−^ mice and homozygous Atp6i^+/+^ mice were infected with *P. gingivalis* (3 mice in each group). The experiment was repeated three times, resulting in a total sample number of N = 9 for each group. The animals were maintained in the University of Alabama at Birmingham (UAB) animal facility and were given distilled water and lab chow ad libitum.

### Infection with *Porphyromonas gingivalis* strains

*Porphyromonas gingivalis* (ATCC: 33277) and *Porphyromonas gingivalis* W50 (ATCC: 53978) were cultured on sheep's blood agar plates supplemented with hemin and vitamin K (BAPHK) for 3 days. For each, a single clone was harvested and transferred to Trypticase Soy Broth supplemented with hemin and vitamin K. On day 4, bacteria were harvested, resuspended, and cell concentrations of each species were determined via optical density readings at 600 nm (One OD unit equals 6.67*10^8^ bacteria). The cell density of each species was adjusted to 10^10^cells/ml in PBS containing 2% carboxymethylcellulose (CMC: Sigma-Aldrich). The periodontal infection regimen was conducted according to a previously described protocol [24], with modifications. In brief, all animals received antibiotic treatment for 3 days to reduce the original oral flora, followed by 3 days of an antibiotic-free period, prior to oral inoculation with 0.2 ml *P. gingivalis* in CMC in 20 µl with a dental micro-brush once per day for 4 consecutive days. To monitor bacterial colonization, the oral cavity of each mouse was sampled on day 14 after *P. gingivalis* infection with a sterile cotton swab, and samples were incubated anaerobically to identify *P. gingivalis* [24].

### AAV-shRNA-Atp6i/TIRC7 transduction of *P. gingivalis* W50 infected mice

We injected AAV-sh-Atp6i in a site-specific manner as described previously with some modification [13]. In brief, mice were anesthetized via peritoneal injection with 62.5 mg/kg ketamine and 12.5 mg/kg xylazine. Starting 4 days after the initial infection and continuing for 5–7 consecutive days, mice were injected approximately 0.3–0.5 mm above the gingival margin of the maxillary molars on the right and left palatal aspects with 3 ul containing 2×10^9^ packaged genomic particles in PBS, of either AAV-sh-Atp6i or AAV-sh-luc-YFP viral vector using 5-μl Hamilton syringe attached to a microinfusion pump (World Precision Instruments, Sarasota, FL). As a negative control (normal), mice were not infected with *P. gingivalis* W50 or treated with either of the viral vectors. As a positive control (disease), *P. gingivalis*-infected mice were not injected with either viral vector. A timeline for bacterial infection and AAV treatment is provided in Supplemental Diagram S1 in File S1.

### Harvest and preparation of tissue samples

Animals were sacrificed by CO_2_ inhalation 55 days after initial infection. The maxillae were hemisected. For bone height measurements, seven samples from the left side were defleshed in 2.6% sodium hypochlorite [25] for 30–40 minutes, rinsed in tap water three times, placed in 70% alcohol, stained with 1% methylene blue, and mounted on microscope slides for bone loss measurements. Three samples from the right side were immediately fixed in 4% paraformaldehyde and prepared for histological analysis according to standard protocol with modifications. In brief, samples for paraffin sections were fixed in 4% formaldehyde for 24 hours, washed with PBS, decalcified in 10% EDTA in 0.1M TRIS solution (PH = 7.0) for 10 days (replenished each day), washed with 1XPBS three times, and embedded in paraffin after series dehydration. Three samples were similarly prepared for frozen section. Basically, samples were fixed in 4% formaldehyde for 24 hours, washed with 1XPBS three times, decalcified in 10% EDTA in 0.1M TRIS solution (PH = 7.0) for 10 days (replenished each day), and washed with PBS for 3 times. Then, the samples were soaked in 30% sucrose for 24 hours, submerged in frozen section compound (FSC 22, Surgipath, Leica Microsystems), and stored at −80°C prior to cryostat sectioning. Four samples from the right side in every independent experiment were obtained for Real-Time quantitative PCR (qRT-PCR) analyses and Enzyme-linked immunosorbent assays (ELISAs). Gingival tissues and/or alveolar bone were isolated under a surgical microscope. Gingival tissues and alveolar bone from four samples were pooled for qRT-PCR, and gingival tissues from another four samples were pooled for ELISAs for cytokines. These experiments were repeated three times.

### Bone loss measurements

The protocol for imaging was carried out as previously described [14]. Briefly, images of molar tooth roots and alveolar bone were captured using digital microscopy. The area of periodontal bone loss was determined using Adobe PhotoshopTM (Adobe Systems, San Jose, CA, USA). In addition, the polygonal area enclosed by the cemento-enamel junction, the lateral margins of the exposed tooth root, and the alveolar ridge was measured using the ImageJ analysis software (Wayne Rasband, NIH, Bethesda, MD, USA). Measurements were expressed in mm^2^.

### Western blotting analysis

Western blotting was performed as previously outlined [17], [26] and visualized and quantified using a Fluor-S Multi-Imager with Multi-Analyst software (Bio-Rad). A rabbit anti-Atp6i antibody previously generated in our lab [3] was used at a 1∶1000 dilution, with goat anti-rabbit IgG-HRP (7074S, Cell signaling) used at a 1∶5000 dilution to visualize the reaction.

### Histological analysis and immunofluorescence analysis

Histological analysis included hematoxylin & eosin (H&E) staining and calculation of the width of the periodontal ligament in each group as described [27]. We performed immunofluorescence analysis in order to examine the effect of AAV-sh-Atp6i treatment on the expression of Atp6i in osteoclasts and on the number of CD3 expressing T-cells *in vivo*. Immunofluorescence analysis was performed as outlined previously [3], with the exception that we used anti-Atp6i [3] and rabbit polyclonal anti-CD3 (Abcam, Cambridge, MA) as the primary antibodies and rhodamine red-X-labeled secondary antibodies (1∶200 dilution). Data was documented using epifluorescence on a Zeiss axioplan microscope in the Developmental Neurobiology Imaging and Tissue Processing Core at the UAB Intellectual and Disabilities Research Center. Nuclei were visualized with 1 ìg/ml DAPI (4′,6-diamidino-2-phenylindole; Sigma). The experiments were carried out in triplicate on three independent occasions.

### Acridine orange staining

Acid production was determined using acridine orange as described previously [3]. Osteoclasts that had been transduced with viral vectors after 1 day of RANKL/M-CSF stimulation were incubated in α-MEM containing 5 ug/ml of acridine orange (Sigma) for 15 minutes at 37°C, washed, and chased for 10 minutes in fresh media. The cells were observed under a fluorescence microscope with a 490 nm excitation filter and a 525 nm arrest filter. The experiment was performed in duplicate on three independent occasions in a 24-well plate.

### *In vitro* bone resorption assays

Bone resorption activity was assessed as described [28] with minor modifications. MBM cells were cultured on bovine cortical bone slices in 24-well plates and transduced with viral vectors after 1 day of RANKL/M-CSF stimulation. The bone slices were harvested after 6 days and the culture media collected. Cells adhering to the bone slices were subsequently removed with 0.25 M ammonium hydroxide and mechanical agitation. Bone slices were subjected to scanning electron microscopy (SEM) using a Philips 515 SEM (Department of Materials Science and Engineering, UAB). We also assessed *In vitro* bone resorption using wheat germ agglutinin (WGA) to stain exposed bone matrix proteins as described [29]. The assays were performed in triplicate. The data were quantified by measuring the percentage of the areas resorbed in three random resorption sites, as determined using ImageJ analysis software.

### Real-time quantitative PCR (qRT-PCR)

To determine the effect of Atp6i knockdown on the levels of regulatory cytokines in inflammatory periodontal tissues, gingival tissues and alveolar bone were isolated and kept at −70°C. The prepared samples were then transferred to a tube containing beads (Nextadvance Inc.) and homogenized using a Blender (Bullet Blender®, Nextadvance). RNA extraction was carried out under standard procedures using Trizol reagent (Invitrogen). The extracted RNA was reverse transcribed using Vilo® Master Kit (Invitrogen). qRT-PCR was performed as described [30], [31] using TaqMan probes purchased from Applied Biosystems as listed in Table 1 according to the manufacturer's instructions.

**Table 1:** qRT-PCR Primer Numbers.

Gene	Applied Biosystems Assay ID
*Acid phosphatase 5* (*Acp5*)	Mm00475698_m1
*T-cell immune response regulator 1* (*Atp6i*)	Mm00469394_m1
*Calcitonin receptor* (*Calcr*)	Mm00432271_m1
*Colony stimulating factor 1 receptor* (*CD115*)	Mm01266652_m1
*Cathepsin K* (*Ctsk*)	Mm00484039_m1
*Tumor necrosis factor (ligand) superfamily, member 11*(*RANKL*)	Mm00441906_m16
*Interleukin 6 (interferon, beta 2*) (*IL-6*)	Mm00446190_m1
*Interleukin 1, alpha* (*IL-1α*)	Mm00439620_m1
*Interleukin 1, beta* (*IL-1β*)	Mm01336189_m1
*Interleukin 17 receptor A* (*IL-17A*)	Mm00439618_m1

Briefly, cDNA fragments were amplified by TaqMan® Fast Advanced Master Mix (Applied Biosystems). Fluorescence from each TaqMan probe was detected using the Step-One real-time PCR system (Applied Biosystems). The mRNA expression level of the housekeeping gene hypoxanthine-guanine phosphoribosyl transferase (*Hprt*) was used as an endogenous control and enabled calculation of specific mRNA expression levels as a ratio of *Hprt (deltaCT)*. PCR was performed under standard conditions and repeated at least three times.

### Enzyme-linked immunosorbent assay (ELISA)

To determine the effect of Atp6i knockdown on the levels of regulatory cytokines in gingival tissues, we used ELISA as previously described [24], [32]. Briefly, assays for cytokines in the tissue extracts used commercially available ELISA kits that were obtained from the following sources: IL-1α (Endogen, Cambridge, MA; sensitivity 6 pg/ml), IL-6 (BioSource International, Camarillo, CA; 8 pg/ml), and IL-17A. All assays were conducted in accordance with the manufacturer's instructions with results expressed as pg cytokine/mg tissue.

### Statistical analysis

Experimental data were reported as mean ± SD. All experiments were performed in triplicate on three independent occasions. *In vitro* osteoclast and bone resorption data were analyzed with the Student's t-test. Bone loss measurements, histological and immunohistochemical measurements, qRT-PCR, and ELISA data were analyzed by ANOVA. P values <0.05 were considered significant. Data quantification analyses were performed using the NIH Image J Program as described [33], [34].

## Results

### AAV-shRNA-Atp6i/TIRC7 simultaneously targeted *Atp6i* and *TIRC7* mRNA and efficiently knocked down the expression of Atp6i

**Figure 1:**
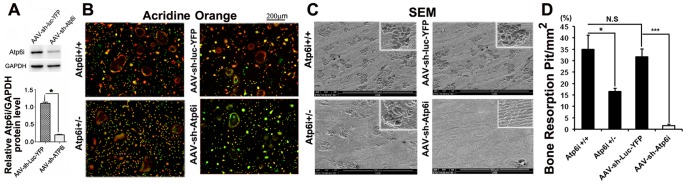
Both knockdown and haploinsufficiency of Atp6i impaired osteoclast-mediated extracellular acidification and bone resorption *in vitro*. (A) Western blot of Atp6i expression in mouse bone marrow (MBM) stimulated with M-CSF/RANKL for 1 day and transduced with AAV-sh-luc-YFP or AAV-sh-Atp6 and cultured for an additional 4 days. Quantification of western blot analysis demonstrates that AAV-sh-Atp6i treated osteoclasts have significantly reduced expression of Atp6i as compared to the AAV-sh-luc-YFP treatment group. (B) Acridine orange staining of osteoclasts including cells without fusion (<3 nuclei). Osteoclasts from Atp6i^+/−^ mice show weak extracellular acidification (weak orange coloration) compared to osteoclasts from Atp6i^+/+^ mice. Osteoclasts transduced with AAV-sh-Atp6i have even less extracellular acidification (green coloration) compared to osteoclasts transduced with AAV-sh-luc-YFP. (C) Bone resorption pits were visualized by scanning electron microscopy (SEM). AAV-sh-Atp6i treatment and Atp6i knockdown greatly reduced the functions of osteoclasts. (D) Quantification of SEM bone pits shows bone resorption in the bone slices is significantly lower in the AAV-sh-Atp6i treatment group as compared to the AAV-sh-luc-YFP treatment group (n = 3 in each group). *** indicates P<0.001.

To enable simultaneous inhibition of inflammation and bone resorption through a single target, we generated shRNA that would simultaneously target *Atp6i* (a subunit of osteoclast specific proton pump) and *TIRC7* (T-cell immune response cDNA 7, an isoform of Atp6i). In order to determine the ability of the AAV-sh-Atp6i (knockdown) and the AAV-sh-luc-YFP (control) vectors to transduce target cells, we examined the expression of EGFP or YFP in target cells. Mouse bone marrow (MBM) isolated from wild-type BALB/cJ mice was cultured with M-CSF and RANKL to generate osteoclasts, that were then transduced with AAV-sh-Atp6i or AAV-sh-luc-YFP. As shown in Fig. S1A in File S1, cell fluorescence indicated that efficient transduction of pre-osteoclasts and osteoclasts with AAV-sh-Atp6i or AAV-sh-luc-YFP were achieved. To confirm the effect of transduction on gene silencing, we performed Western blot analysis and found that osteoclasts transduced with AAV-sh-Atp6i had an 80% reduction in Atp6i expression compared to osteoclasts transduced with AAV-sh-luc-YFP (Fig. 1A). Taken together, our results indicate that AAV-sh-Atp6i, which targets Atp6i and its isoform TIRC7, efficiently reduces Atp6i protein expression *in vitro*.

### Depletion of *Atp6i* potently inhibited extracellular acidification by osteoclasts and osteoclast-mediated bone resorption *in vitro*

Atp6i is an osteoclast-specific proton pump subunit essential for osteoclast-mediated extracellular acidification in bone resorption [3]. Through acridine orange staining, we found that both osteoclasts transduced with AAV-sh-Atp6i and osteoclasts from Atp6i^+/−^ mice showed weak extracellular acidification compared to osteoclasts transduced with AAV-sh-luc-YFP (control) or osteoclasts from Atp6i^+/+^ mice (Fig. 1B). Accordingly, we anticipated that AAV-sh-Atp6i would affect bone resorption by osteoclasts. Compared to control osteoclasts, AAV-mediated knockdown of Atp6i completely inhibited bone resorption (Fig. 1C, D and Fig. S1B, C in File S1). Depletion of *Atp6i* in Atp6i^+/−^ mutant mice partially inhibited bone resorption mediated by osteoclasts (Fig. 1C, D). Atp6i^+/−^ mice were used a potential genetic control for this knockdown study since Atp6i^−/−^ mice die within 3 weeks of birth. These data demonstrate that AAV-sh-Atp6i is a powerful inhibitor of extracellular acidification by osteoclasts and osteoclast-mediated bone resorption *in vitro*.

### AAV effectively transduced periodontal tissue and displayed a local distribution pattern *in vivo*

**Figure 2:**
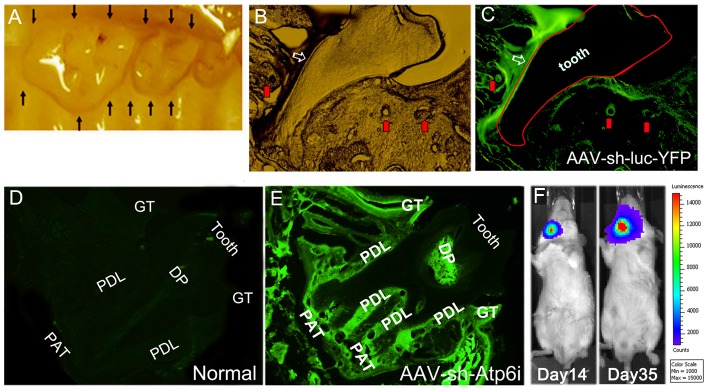
AAV effectively transduced periodontal tissue and displayed a local distribution pattern *in vivo*. (A) Mouse maxillary molars showing sites of AAV injection sites (black arrows). (B, C) The light microscope image (B) and fluorescence microscope image (C) reveal YFP expression after local injection with AAV-sh-luc-YFP. (D, E) Compared to normal mouse tissues that did not receive AAV treatment (D), local injection with AAV-sh-Atp6i resulted in EGFP expression (E) in gingival tissue (GT), periodontal ligament (PDL), dental pulp (DP), periapical tissue (PAT), and alveolar bone adjacent to PDL. (F) Image analysis 14 or 35 days after local injection of AAV-Luc into the mouse lower right jaw; luciferase expression was sustained and limited to the local injection site. (n = 5 for each time point, each time point was repeated 3 times).

To determine if our AAV vectors could effectively transduce periodontal tissues *in vivo*, we examined expression of EGFP or YFP following injection of AAV-sh-Atp6i or AAV-sh-luc-YFP into gingival tissue on the right side of the maxilla in twelve locations as indicated in Figure 2A. Local injection of AAV-sh-luc-YFP *in vivo* transduced periodontal tissues (Fig. 2B). Following local injection with AAV-sh-Atp6i, EGFP expression was observed in gingival tissue, periodontal ligament, dental pulp, and periapical tissue, indicating that AAV-sh-Atp6i effectively diffuses throughout periodontal tissues (Fig. 2D, E). To further evaluate the potential of AAV vectors as tools for gene transfer to periodontal tissues, we assessed the ability of AAV-luc to achieve sustained and localized luciferase expression (Fig. 2F). We found that even 14 or 35 days post AAV-Luc injection, luciferase expression was limited to the local injection site (Fig. 2F). Surprisingly, luciferase expression was higher 35 days post AAV-Luc injection compared to day 14, indicating that injection of AAV vectors into gingival tissue enables sustained synthesis of therapeutic gene products for more than 35 days. These data demonstrate that our AAV-mediated gene therapy is able to effectively transduce periodontal tissue and that injection of AAV into gingival tissue results in localized and sustained synthesis of gene products.

### Atp6i depletion protected mice from bone loss in *P. gingivalis* W50-stimulated periodontitis

**Figure 3:**
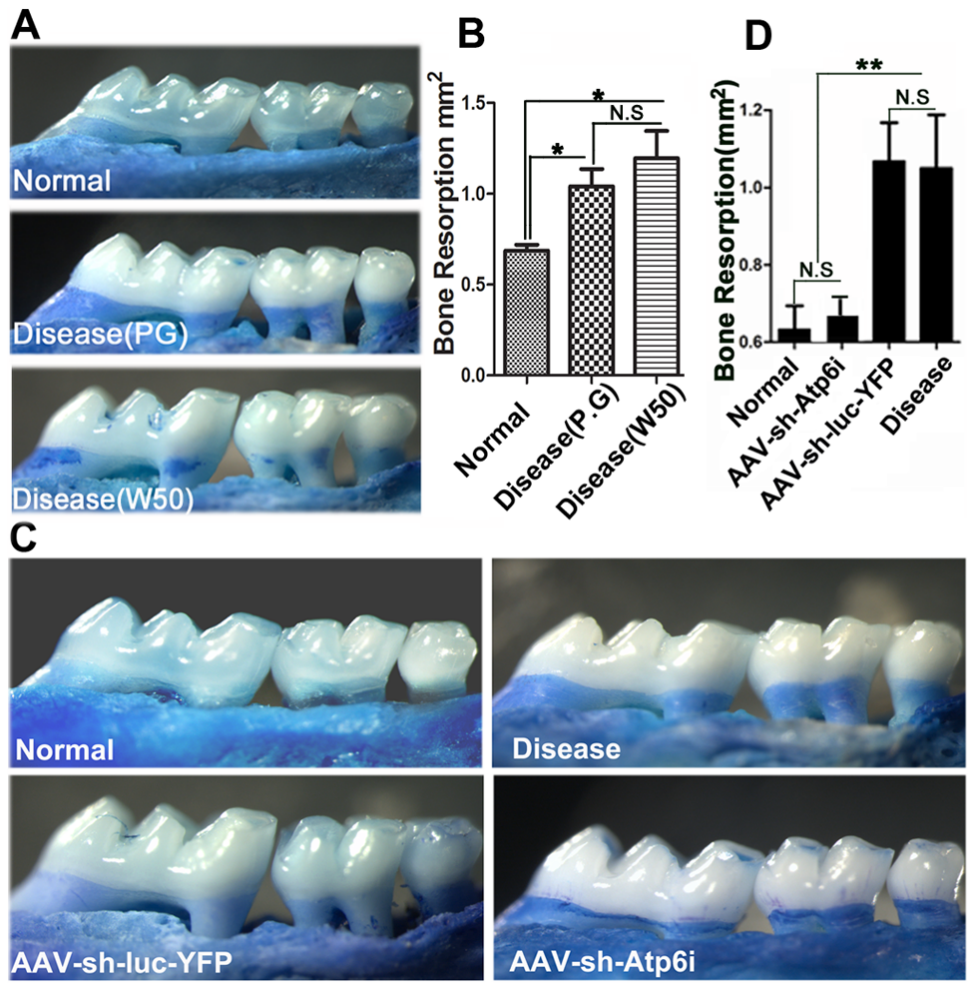
Atp6i knockdown reduced bone resorption in *P. gingivalis* W50-stimulated periodontitis. (A) Representative figures from periodontal disease induced by *P. gingivalis* and *P. gingivalis* W50. (B) There was no significant difference between *P. gingivalis* group (N = 21, n = 7 per group, repeated 3 times) and *P. gingivalis* W50 group (N = 21, n = 7 per group, repeated 3 times). (C) Representative figures from periodontal disease as indicated by alveolar bone loss and root exposure examined in WT BALB/cJ mice that did not receive *P. gingivalis* W50 infection nor any form of treatment (Normal) versus untreated *P. gingivalis* W50-infected mice (disease control) and *P. gingivalis* W50-infected mice treated with AAV-sh-Atp6i or AAV-sh-luc-YFP as (positive control). (D) There was no significant difference in bone loss between the normal group (N = 21, n = 7 per group, repeated 3 times) and the AAV-sh-Atp6i treatment group (N = 21, n = 7 per group, repeated 3 times) (p>0.05). In addition, the AAV-sh-Atp6i treatment group (N = 21, n = 7 per group, repeated 3 times) had significantly less bone loss compared to the AAV-sh-luc-YFP treatment group (N = 21, n = 7 per group, repeated 3 times) and disease control group (N = 21, n = 7 per group, repeated 3 times) (p<0.05).

**Figure 4:**
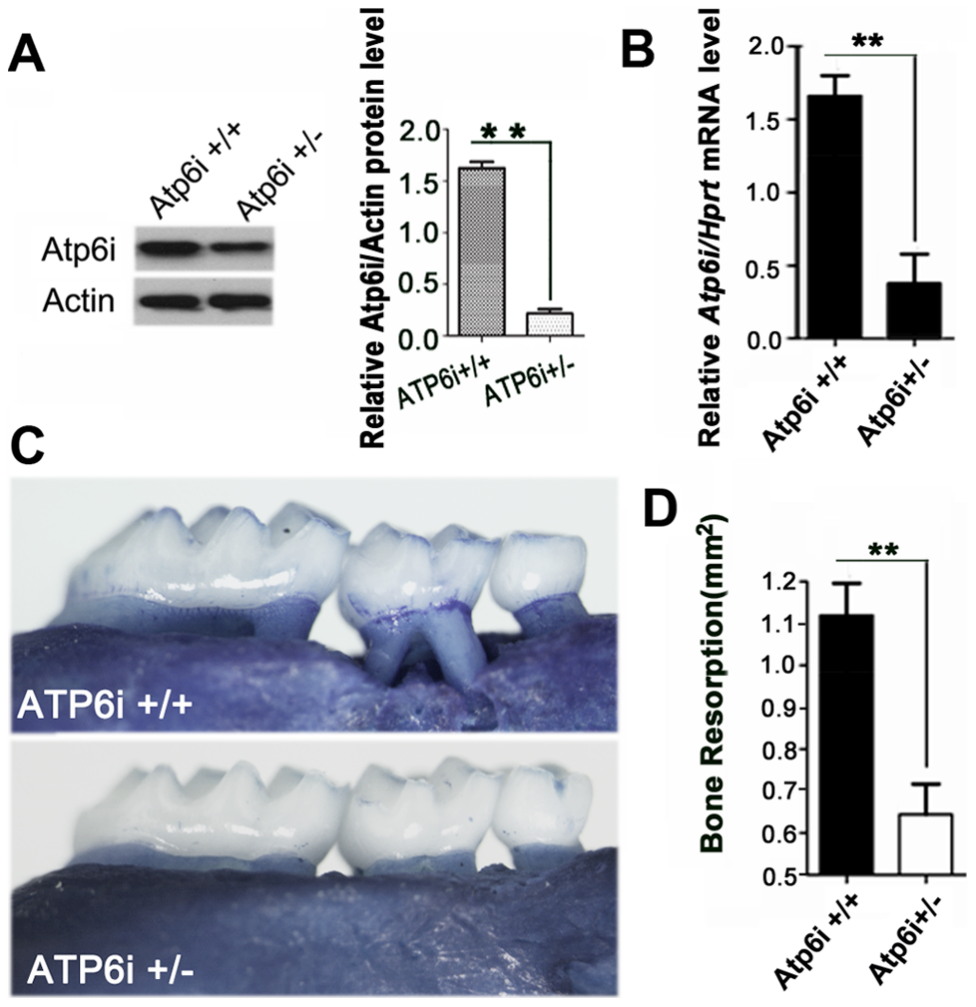
Atp6i depletion protected mice from bone loss in *P. gingivalis* W50-stimulated periodontitis. (A) Western blot illustrates that Atp6i expression is significantly decreased in osteoclasts from Atp6i^+/−^ mice compared to that of Atp6i^+/+^ mice. * indicates P<0.01. (B) qRT-PCR of Atp6i in the periodontal tissues from Atp6i^+/+^ mice and Atp6i^+/−^ mice. (C) As a genetic control to our knockdown study, alveolar bone loss and root exposure was also examined in Atp6i^+/−^ mice and Atp6i^+/+^ mice infected by *P. gingivalis* W50. (D) Atp6i^+/−^ mice (N = 9, n = 3 per group, repeated 3 times) showed significantly (P = 0.011) less *P. gingivalis* W50-induced alveolar bone resorption and root exposure than Atp6i^+/+^ mice (N = 9, n = 3 per group, repeated 3 times).

The *P. gingivalis* (*P. gingivalis*) strains have been shown to induce significant bone loss in animal models of human periodontal disease. We first determined the bone resorption effects induced by these two strains, the result showed that both *P. gingivalis and P. gingivalis* W50 can induce significant bone loss compared with normal (P<0.05) (Fig. 3A, B). There was no significant difference in the bone loss area between these two strains (Fig. 3A, B). To determine if Atp6i knockdown by local injection protects mice from *P. gingivalis*-stimulated bone loss, we analyzed the degree of alveolar bone loss in uninfected BALB/cJ mice (normal) compared to untreated *P. gingivalis* W50-infected mice (disease) and *P. gingivalis* W50-infected mice treated with AAV-sh-Atp6i or with AAV-sh-luc-YFP as a control (Fig. 3C). AAV-sh-Atp6i or AAV-sh-luc-YFP was locally injected into the gingival tissue one day after four consecutive days of *P. gingivalis* W50 administration. There was no significant difference in bone loss between the uninfected normal group and the AAV-sh-Atp6i treatment group (p>0.05) (Fig. 3C, D). However, the disease control group and the AAV-sh-luc-YFP treatment group had significantly more bone loss compared to the AAV-sh-Atp6i group (p<0.05) or the normal group (p<0.0001) (Fig. 3C, D). Indeed, AAV-mediated *Atp6i/TIRC7* knockdown largely protected mice from *P. gingivalis* W50infection-stimulated bone erosion (>85%) (Fig. 3C, D)). To confirm that heterozygous Atp6i^+/−^ mice can serve as a genetic control for this knockdown study, we performed Western blot analysis and determined that the level of Atp6i protein was significantly reduced in heterozygous Atp6i^+/−^ mice compared to homozygous Atp6i^+/+^ mice (Fig. 4A). Atp6i^+/−^ mice and Atp6i^+/+^ mice were also infected by *P. gingivalis* W50 (Fig. 4C). *P. gingivalis* W50-infected Atp6i^+/+^ mice had significantly more alveolar bone resorption and root exposure than *P. gingivalis* W50-infected Atp6i^+/−^ mice (Fig. 4C, D). These results indicate that Atp6i depletion protects against *P. gingivalis* W50-stimulated bone erosion in the mouse model of periodontal disease.

### AAV-mediated Atp6i knockdown decreased bone erosion and the number of T-cells in the periodontal area

**Figure 5:**
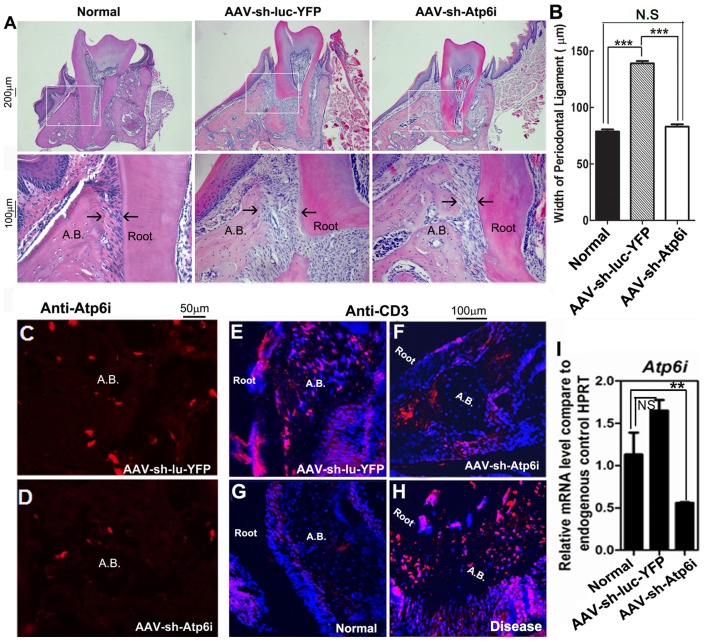
AAV-mediated Atp6i knockdown decreased bone erosion and the number of T-cells in the periodontal area. (A) Representative figures from hematoxylin & eosin (H&E) staining of sections from uninfected mice (normal) or *P. gingivalis* W50-infected mice treated with AAV-sh-Atp6i or AAV-sh-luc-YFP (control). (B) The distance between the tooth root surface and the alveolar bone is almost doubled in the control AAV-sh-luc-YFP treatment group (N = 9, n = 3 per group, repeated 3 times) compared to the normal (N = 9, n = 3 per group, repeated 3 times) and AAV-sh-Atp6i treatment groups (N = 9, n = 3 per group, repeated 3 times), indicating that AAV-sh-Atp6i prevents alveolar bone loss. (C, D) Representative figures from immunofluorescence staining of Atp6i (red) reveals that AAV-sh-Atp6i treatment reduces expression of Atp6i *in vivo*. (E–H) Representative figures from immunofluorescence staining of alveolar sections indicates that uninfected mice (normal) (N = 12, n = 4 per group, repeated 3 times) and *P. gingivalis* W50-infected mice treated with AAV-sh-Atp6i (N = 12, n = 4 per group, repeated 3 times) have less CD3 positive (red) T-cells compared to untreated *P. gingivalis* W50-infected mice (disease) (N = 12, n = 4 per group, repeated 3 times) or *P. gingivalis* W50-infected mice treated with AAV-sh-luc-YFP (N = 12, n = 4 per group, repeated 3 times). Cell nuclei were labeled using DAPI DNA stain (blue). (I) qRT-PCR of Atp6i in the periodontal tissues from normal mice or infected mice treated with AAV-sh-luc-YFP or with AAV-sh-Atp6i (pooled 4 samples each time in each group on three independent experiments). Expression levels were normalized to the housekeeping gene hypoxanthine-guanine phosphoribosyl transferase (Hprt). A.B. indicates alveolar bone. ** indicates P<0.01, *** indicates P<0.001, N.S. indicates P>0.05.

In order to determine if the periodontal ligament between the tooth root and the bone is normalized by AAV-sh-Atp6i treatment, we examined hematoxylin & eosin-stained sections from uninfected (normal) mice and *P. gingivalis* W50-infected mice treated with AAV-sh-Atp6i or AAV-sh-luc-YFP (positive control). It was determined that the distance between the tooth root surface and the alveolar bone is almost doubled in the control AAV-sh-luc-YFP treatment group compared to the normal and AAV-sh-Atp6i treatment groups (Fig. 5A, B), indicating that AAV-sh-Atp6i prevents periodontal ligament damage and alveolar bone loss. In order to verify that AAV-sh-Atp6i reduces expression of Atp6i in osteoclasts *in vivo*, tooth root sections from *P. gingivalis* W50-infected mice treated with AAV-sh-Atp6i or AAV-sh-luc-YFP were subjected to immunofluorescent staining using Atp6i antibody (Fig. 5C, D). We found that AAV-sh-Atp6i treatment notably reduced expression of Atp6i *in vivo* (Fig. 5C, D). To investigate the effect of AAV-sh-Atp6i on T-cells *in vivo*, alveolar sections were subjected to immunofluorescent staining using CD3 antibody to detect T-cells (Fig. 5E–H). Our results showed that the number of CD3 expressing T-cells in the periodontal ligament was significantly reduced in the AAV-sh-Atp6i treatment group (Fig. 5F) compared to that of the AAV-sh-luc-YFP treatment group (Fig. 5E) or disease control group (Fig. 5H). Real-Time quantitative PCR (qRT-PCR) analysis further demonstrated that AAV-sh-Atp6i effectively knocks down the expression level of Atp6i in periodontal tissues (Fig. 5I). These results indicate that AAV-sh-Atp6i not only protects mice from *P. gingivalis* W50-stimulated periodontal ligament widening and bone erosion, but that AAV-sh-Atp6i effectively reduced Atp6i expression *in vivo* and protects against inflammation caused by periodontal disease.

### AAV-sh-Atp6i reduced the expression of osteoclast marker genes and cytokines in the periodontal tissues back toward normal expression levels

**Figure 6:**
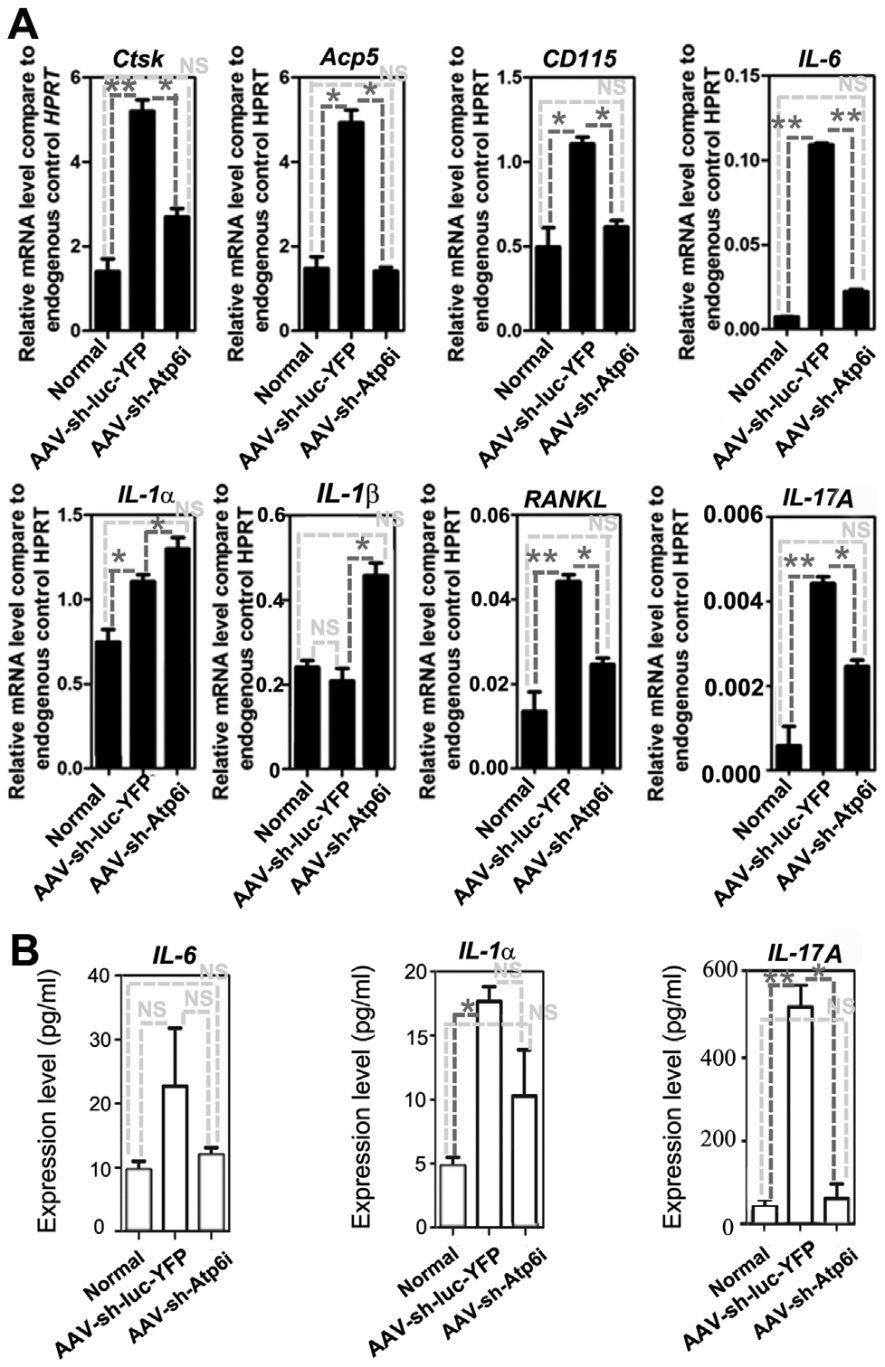
AAV-sh-Atp6i reduced the expression of osteoclast marker genes and cytokines in the periodontal tissues. (A) qRT-PCR of osteoclast-specific functional genes (*i.e. Ctsk* and *Acp5*), genes important for osteoclast differentiation (*i.e. RANKL*), *a* gene common to macrophages and osteoclasts (*i.e. CD115*), and cytokines (*i.e. IL-6, IL-1α, IL-1β,* and *IL-17A*) in the periodontal tissues of uninfected mice (normal) or *P. gingivalis* W50-infected mice treated with AAV-sh-luc-YFP or with AAV-sh-Atp6i. Expression levels were normalized to the housekeeping gene hypoxanthine-guanine phosphoribosyl transferase (Hprt) (pooled 3 samples each time in each group on three independent experiments). (B) IL-6, IL-1α, and IL-17A levels in the periodontal tissues as detected by ELISA (pooled 3 samples each time in each group on three independent experiments). * indicates P<0.05, ** indicates P<0.01, N.S. indicates P>0.05.

To investigate the effect of AAV-sh-Atp6i on the interplay between bone resorption and inflammation, qRT-PCR was used to quantify mRNA expression in the periodontal tissues (Fig. 6A). Compared to normal uninfected mice, we found that genes important for osteoclast differentiation [*i.e. RANKL* and Interleukin-6 (*IL-6*)] and osteoclast-specific functional genes [*i.e.* Cathepsin K (*Ctsk)* and acid phosphatase 5 tartrate resistant (*Acp5*)] increased in expression in *P. gingivalis* W50-infected mice treated with the control AAV vector AAV-sh-luc-YFP (Fig. 6A). Importantly, AAV-sh-Atp6i treatment was able to reduce the expression of *RANKL*, *IL*-6, *Ctsk*, and *Acp5* in *P. gingivalis* W50-infected mice such that it resembled the expression level in normal uninfected mice (Fig. 6A). AAV-sh-Atp6i treatment similarly reduced the expression of colony stimulating factor 1 receptor (*CD115* or *M-CSF-R*), which is important for both macrophage and osteoclast differentiation (Fig. 6A). In addition, AAV-sh-Atp6i treatment of *P. gingivalis* W50-infected mice resulted in decreased expression of T-cell-derived inflammatory cytokine *IL-17A* (Fig. 6A). These results were confirmed at the protein level by Enzyme-linked immunosorbent assay (ELISA) of the level of IL-6 and IL-17A in inflammatory gingival tissue (Fig. 6B). Additional analysis is required to determine the effect of Atp6i knockdown on bone resorptive cytokines *IL-1α* and *IL-1β* since qRT-PCR analysis indicates that AAV-sh-Atp6i treatment increases expression of *IL-1α* and *IL-1β* in *P. gingivalis* W50-infected mice, while ELISA indicates that AAV-sh-Atp6i treatment decreases expression of *IL-1α* (Fig. 6A, B). These results indicate that Atp6i knockdown reduces expression of genes important for osteoclastic bone resorption and inflammation in periodontal tissues and that bone resorptive cytokines *IL-1α* and *IL-1β* may be part of an alternative signaling pathway.

## Discussion

Periodontal disease is an infection with a bacterial complex which prominently includes the pathogen *Porphyromonas gingivalis*, which has been associated with disease progression [35]. In periodontitis, host immune and inflammatory responses play a key role [15], [24], by inducing soft tissue damage as well as activating osteoclast-mediated alveolar bone resorption [36]. In the present study, we demonstrated that gene knockdown with an AAV vector that targeted the shared sequences of *Atp6i*, a component of the osteoclast proton pump, and its isoform *TIRC7*, a regulator of T-cell activation, simultaneously inhibited both molecules and dramatically reduced periodontal disease progression. The AAV-sh-Atp61 vector impaired osteoclastic bone resorption *in vitro* through inhibition of extracellular acidification (Fig. 1), and furthermore reduced proinflammatory cytokine expression, osteoclast number, and protected mice from *Porphyromonas gingivalis*-stimulated periodontal bone loss *in vivo* by 80%. These studies thus demonstrate that local AAV-sh-Atp6i gene therapy efficiently protects against periodontal tissue damage and alveolar bone loss, and may represent a powerful new treatment strategy for this disease.

### Local delivery of AAV-sh-Atp6i gene therapy is a promising therapeutic tool for periodontal disease

Current clinical therapies for periodontal disease have been focused on anti-microbial treatments as well as surgery, which generally have limited efficacy [37]. In contrast, gene therapy has the potential to achieve an outcome that no other technology can accomplish by enabling sustained, endogenous, synthesis of therapeutic gene products in a relatively non-invasive manner [36]. Which genes are the best to target and which vectors can be safely employed are fundamental questions that must be rigorously addressed in order to develop a practical therapeutic modality that is both effective and safe [36]. In terms of targets, our group first characterized Atp6i [38] and demonstrated that it is critical for the extracellular acidification that is necessary for bone resorption. Atp6i is relatively specific to the osteoclast proton pump [3], [4], and its abrogation results in complete loss of bone resorptive ability [3]. The Atp6i isoform TIRC7 is a regulator of T-cell function, and its inhibition prevents acute allograft rejection [5], [7], [9]. Atp6i^+/−^ mice, which served as a genetic control for this knockdown study, also demonstrated that depletion of Atp6i protects mice from *P. gingivalis*-stimulated bone loss. Accordingly, we generated a vector that expressed shRNA that would simultaneously target exon 15 of Atp6i and exon 10 of TIRC7, to take advantage of the synergistic functions of these two molecules/cell types in periodontal disease pathogenesis.

Gene transfer with AAV2 has been shown to be safe and effective in a number of disease models. Self-complementary vectors derived from AAV2 were used *in vivo* to knock down ERα expression in the brain [13]. Intra-articular injection of AAV2 resulted in sustained gene expression for at least 120 days [39]. In bone resorption models, osteoprotegerin (OPG) gene transfer attenuated osteoclast number and activity in debris-induced osteolysis [40], and reduced aseptic joint prosthesis loosening [41]. In a non-oral infection model, AAV-mediated gene transfer of IL-10 reduced airway inflammation in mice infected with *Pseudomonas aeruginosa*, the major pathogen in cystic fibrosis [42]. Collagen-induced arthritis and inflammatory responses were attenuated by AAV-IL-4 [43].

In periodontal disease, AAV2 achieved the highest transduction rates for human primary periodontal ligament cells as well as periodontal tissues [44]. Systemic transduction with TIMP-4 was reported to reduce both adjuvant-induced arthritis and periodontitis in rats [45]. Inhibition of periodontal bone loss was also reported following AAV gene transfer of mitogen-activated protein kinase phosphatase-1 (MKP-1), which dephosphorylates MAPKs and inhibits immune responses [46]. Transduction of gingival tissues with AAV/2/1-TNF Receptor:Fc reduced *P. gingivalis*-induced periodontal bone loss by approximately 50% in a mouse model, with concomitant reductions in proinflammatory cytokines and osteoclasts [47]. Of interest, in the latter study the AAV-TNFR:Fc vector was delivered at multiple gingival sites three times per week for 8 weeks, compared to the single time point injections of AAV-sh-Atp6i in the present study. The greater efficiency of Atp6i/TIRC7 inhibition (80%) may reflect the critical importance and synergistic function of these two molecules in periodontal disease pathogenesis, one critical for osteoclastic resorption and the other for T cell-mediated inflammatory processes that induce tissue destruction as well as osteoclast formation. In general, compared to AAV vectors that separately target resorption or inflammation, the dual functionality of AAV-sh-Atp6i appears to be considerably more efficient. Another factor may be that local injection of AAV-sh-Atp6i into the periodontal tissue resulted in persistence of the vector for at least 35 days (Fig. 2), indicating sustained synthesis of therapeutic inhibitory RNA in the gingival sites. Local delivery of gene therapy is favored over systemic administration since local delivery restricts the synthesis of therapeutic gene products to selected sites, which minimizes exposure of non-target tissues and reduces side effects and treatment costs [36]. AAV-sh-Atp6i not only reduces bone loss (Fig. 3) and inflammation in periodontitis, but it is also inhibits periapical bone erosion and inflammation in the mouse model of endodontic disease [48] and may be useful for other diseases involving bone resorption and the immune system.

### Simultaneously targeting Atp6i and its TIRC7 isoform allows for increased efficiency in the treatment of inflammation and bone loss associated with periodontal disease

Gene therapy with AAV-sh-Atp6i also reduced the number of T-cells in the periodontal tissues of *P. gingivalis*-infected mice, and also decreased the expression of osteoclast-specific functional genes *Acp5* and *Ctsk* reflecting the lower number of osteoclasts in tissues. Interestingly, osteoclasts derived from Atp6i^+/−^ mutant mice still maintain about 40% bone resorption activity (Fig. 1C, D), however Atp6i haploinsufficiency dramatically protected mice from bone loss *in vivo* in the *P. gingivalis* W50-stimulated periodontitis model (Fig. 4C, D). Atp6i haploinsufficiency may protect mice from bone loss in the *P. gingivalis* W50-stimulated periodontitis model through both reduced bone resorption and reduced inflammation. Our ELISAs and qRT-PCR analyses also showed a decrease in inflammation in the AAV-sh-Atp6i treatment group. The reduction in inflammation in the AAV-sh-Atp6i treatment group may be partially due to the downregulation of *CD115* (important for monocyte and macrophage differentiation), and IL-6 (a proinflammatory cytokine associated with periodontal disease). Notably, IL-6, which is secreted by osteoblasts in response to bone resorbing agents (*e.g.*IL-1 and TNF-α) [49], promotes both inflammation and bone resorption [50]. Thus, AAV-sh-Atp6i inhibits both processes not only through reduced expression of Atp6i, but also through reduced inflammatory cytokine expression. Due to the limited tissue available for ELISA assays, we chose to focus our examination on the expression changes of inflammatory factors since it would give more mechanism information. Consistent with the reduced number of T-cells in the periodontal area of *P. gingivalis*-infected mice after AAV-sh-Atp6i treatment (Fig. 5), AAV-sh-Atp6i also results in decreased expression of T-cell-derived inflammatory cytokine *IL-17A* (Fig. 6). This result is also consistent with a previous study reporting that T-cell proliferation was affected in TIRC7 knockout mice [7]. Our study shows that *IL-17A* mRNA expression is very low, while the IL-17A protein level is quite high (Fig. 6). Han *et al*. similarly reported a difference in mRNA expression and protein expression for tumor necrosis factor-a (TNF-a) [51]. Cytokine mRNA expression may not be identical to cytokine protein production due to regulation at the transcriptional, posttranscriptional, and translational levels. Although further study is required to elucidate the relationship between Atp6i/TIRC7 and the bone resorptive cytokines IL-1α and IL-1β, our results indicate that knockdown interferes with positive feedback circuits between bone resorptive and inflammatory processes related to the progression of periodontal disease [52].

### AAV-sh-Atp6i is a promising therapeutic agent for periodontal disease and associated systemic conditions

It has been estimated that approximately 80% of American adults have some form of periodontal disease [53]. Periodontal disease not only leads to the loss of teeth, but has been associated in epidemiologic studies with atherosclerosis, coronary artery disease, premature birth, and stroke, presumably by contributing to overall levels of systemic inflammation [44]. Oral microorganisms have also been linked to infections of the endocardium, meninges, mediastinum, vertebrae, hepatobiliary system, and prosthetic joints [44]. Furthermore, periodontal disease impairs glycemic control in diabetics, and the dominant cause of nursing home–acquired pneumonia is aspiration of oropharyngeal (including periodontal) pathogens [44]. Given that periodontitis is widespread and the mounting scientific evidence of the linkages between chronic oral infections and other health conditions, there is an urgent need for more effective treatments for this disease. Although AAV-vector mediated human gene therapy is still controversial due to potential side effects, this approach is in clinical trials for a number of human diseases [54], and it is considered one of the safest gene therapy approaches. However, its use in the treatment of periodontal disease should continue to be evaluated.

## Supporting Information

File S1 (DOCX)
