# Correction: RNAi-Mediated Silencing of Atp6i and Atp6i Haploinsufficiency Prevents Both Bone Loss and Inflammation in a Mouse Model of Periodontal Disease

**DOI:** 10.1371/journal.pone.0301147

**Published:** 2024-03-20

**Authors:** Hongbing Jiang, Wei Chen, Guochun Zhu, Lijie Zhang, Byron Tucker, Liang Hao, Shengmei Feng, Hongliang Ci, Junqing Ma, Lin Wang, Philip Stashenko, Yi-Ping Li

A duplicate of the Fig 1C Atp6i^+/+^ panel [1] was erroneously used to represent the AAV-sh-luc-YFP results due to an error in figure preparation.

With this correction, the authors provide an updated version of Fig 1 and the underlying raw image data for panel 1C (S2 File). The authors stated that the data underlying Fig 1D are available upon reasonable request. The authors stated that the underlying data for Figs 2–6 and Fig S1 are no longer available.

The authors apologize for the error in the published article.

**Fig 1:**
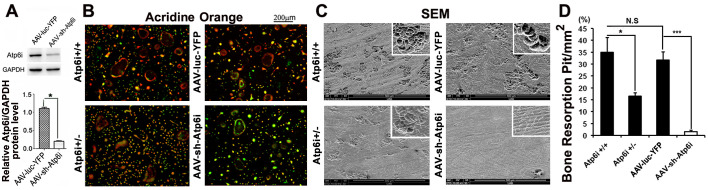
Both knockdown and haploinsufficiency of Atp6i impaired osteoclast-mediated extracellular acidification and bone resorption *in vitro*. (A) Western blot of Atp6i expression in mouse bone marrow (MBM) stimulated with M-CSF/RANKL for 1 day and transduced with AAV-sh-luc-YFP or AAV-sh-Atp6 and cultured for an additional 4 days. Quantification of western blot analysis demonstrates that AAV-sh-Atp6i treated osteoclasts have significantly reduced expression of Atp6i as compared to the AAV-sh-luc-YFP treatment group. (B) Acridine orange staining of osteoclasts including cells without fusion (<3 nuclei). Osteoclasts from Atp6i^+/−^ mice show weak extracellular acidification (weak orange coloration) compared to osteoclasts from Atp6i^+/+^ mice. Osteoclasts transduced with AAV-sh-Atp6i have even less extracellular acidification (green coloration) compared to osteoclasts transduced with AAV-sh-luc-YFP. (C) Bone resorption pits were visualized by scanning electron microscopy (SEM). AAV-sh-Atp6i treatment and Atp6i knockdown greatly reduced the functions of osteoclasts. (D) Quantification of SEM bone pits shows bone resorption in the bone slices is significantly lower in the AAV-sh-Atp6i treatment group as compared to the AAV-sh-luc-YFP treatment group (n  =  3 in each group). *** indicates P<0.001.

## Supporting information

S2 File Fig 1C underlying images. (TIF)
