# Supplementary material for: RNAi-Mediated Silencing of Atp6i and Atp6i Haploinsufficiency Prevents Both Bone Loss and Inflammation in a Mouse Model of Periodontal Disease
Source: PLoS One. 2013 Apr 5;8(4):e58599. doi: 10.1371/journal.pone.0058599 (PMC3618217; doi:10.1371/journal.pone.0058599)
Supplement: File S1 — (DOCX) [file pone.0058599.s001.docx]

**Supplemental Information**

**RNAi-mediated silencing of Atp6i and Atp6i haploinsufficiency prevents both bone loss and inflammation in a mouse model of periodontal disease.**

**Supplemental Data**


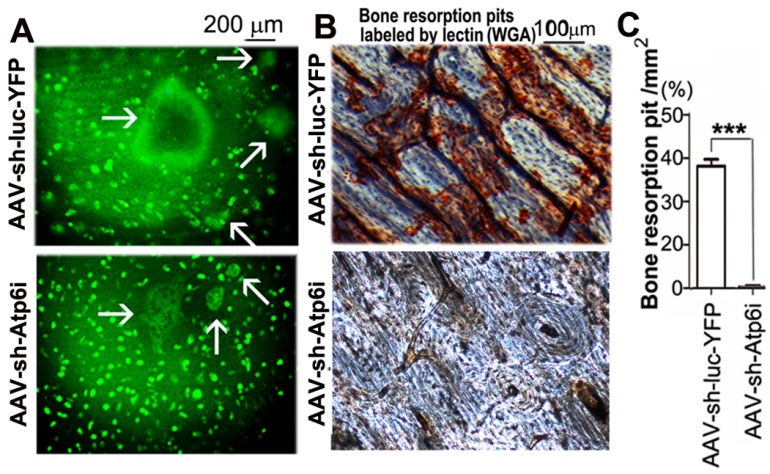


**Supplemental Figure S1. AAV-sh-Atp6i efficiently knocked down the expression of Atp6i and impaired osteoclast-mediated bone resorption *in vitro.*** Mouse bone marrow (MBM) was stimulated with M-CSF/RANKL for 3 days to allow differentiation of osteoclasts that were then transduced with AAV-sh-luc-YFP (control vector) or AAV-sh-Atp6i. (A) Immunofluorescent pictomicrograph of AV-sh-luc-YFP and AAV-sh-Atp6i treatment groups day 7 after transduction. Fluorescence indicates effective transduction of pre-osteoclasts and osteoclasts (white arrows). (B) Bone resorption pits were visualized by wheat germ agglutinin (WGA). (C) Quantification shows that bone resorption in the bone slices is significantly lower in the AAV-sh-Atp6i treatment group compared to the AAV-sh-luc-YFP treatment group (n=3 in each group). *** indicates P < 0.001.

**Supplemental Materials and Methods**

| **Supplemental Table S1. Description of the AAV-shRNA-Atp6i/TIRC7 treatment group**  **and experimental control groups using BALB/cJ mice**  **with and without *P. gingivalis* W50 infection** | | | | |
| --- | --- | --- | --- | --- |
| **Group** | **Number of mice** | **Bacteria Strain** | **Dose of Bacteria** | **AAV Injection** |
| Normal Group | 7 | No Infection | No Infection | No AAV Injection |
| Disease Control Group  (Bacterial infection and PBS) | 7 | *P. gingivalis* W50 | 10^10^cells/ml | No AAV Injection |
| Treatment Group  (Bacterial infection and AAV-shRNA-Atp6i treatment) | 7 | *P. gingivalis* W50 | 10^10^cells/ml | AAV-shRNA-Atp6i/TIRC7 |
| Negative Control Group  (Bacterial infection and AAV-sh-luc-YFP treatment) | 7 | *P. gingivalis* W50 | 10^10^cells/ml | AAV-sh-luc-YFP |

| **Supplemental Table S2. Description of heterozygous Atp6i^+/-^ and homozygous Atp6i^+/+^ groups infected with *P. gingivalis*** | | | | |
| --- | --- | --- | --- | --- |
| **Group** | **Number of mice** | **Bacteria Strain** | **Dose of Bacteria** | **AAV Injection** |
| Heterozygous Atp6i^+/-^ mice | 3 | *P. gingivalis* W50 | 10^10^cells/ml | No AAV Injection |
| Homozygous Atp6i^+/+^ mice | 3 | P. gingivalis W50 | 10^10^cells/ml | No AAV Injection |

**
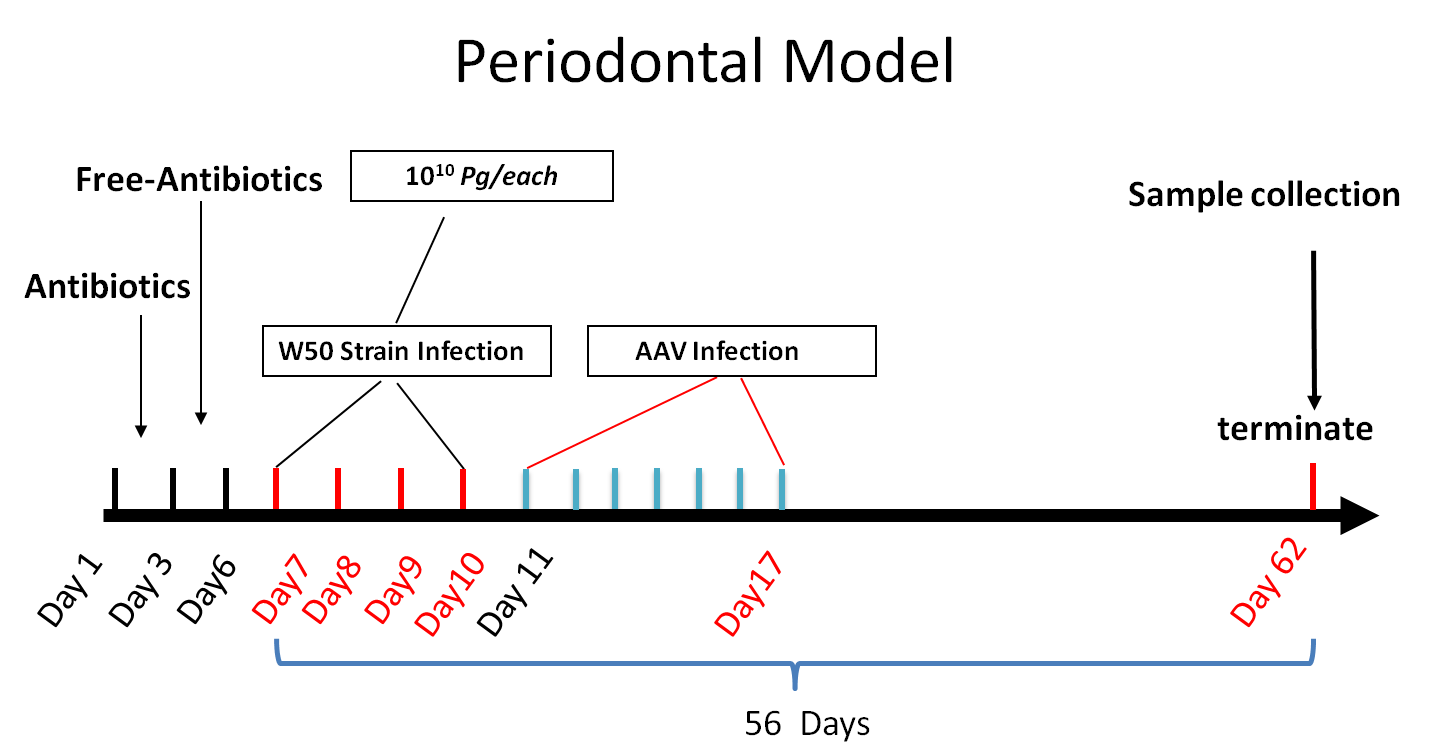
**

**Supplemental Diagram S1. Timeline for bacterial infection and AAV treatment.** All animals received antibiotic treatment for 3 days to reduce the original oral flora, followed by 3 days of an antibiotic-free period, prior to oral inoculation with *P. gingivalis* once per day for 4 consecutive days. Starting 4 days after the initial infection and continuing for 5-7 consecutive days, mice were injected with either AAV-sh-Atp6i or AAV-sh-luc-YFP. The infection period started on the first day of bacterial infection and lasted for 56 days. On day 62, samples were collected for analyses. The total experiment lasted 62 days.
