# Supplementary figures and images for: Correction: RNAi-Mediated Silencing of Atp6i and Atp6i Haploinsufficiency Prevents Both Bone Loss and Inflammation in a Mouse Model of Periodontal Disease
Source: PLoS One. 2024 Mar 20;19(3):e0301147. doi: 10.1371/journal.pone.0301147 (PMC10954136; doi:10.1371/journal.pone.0301147)

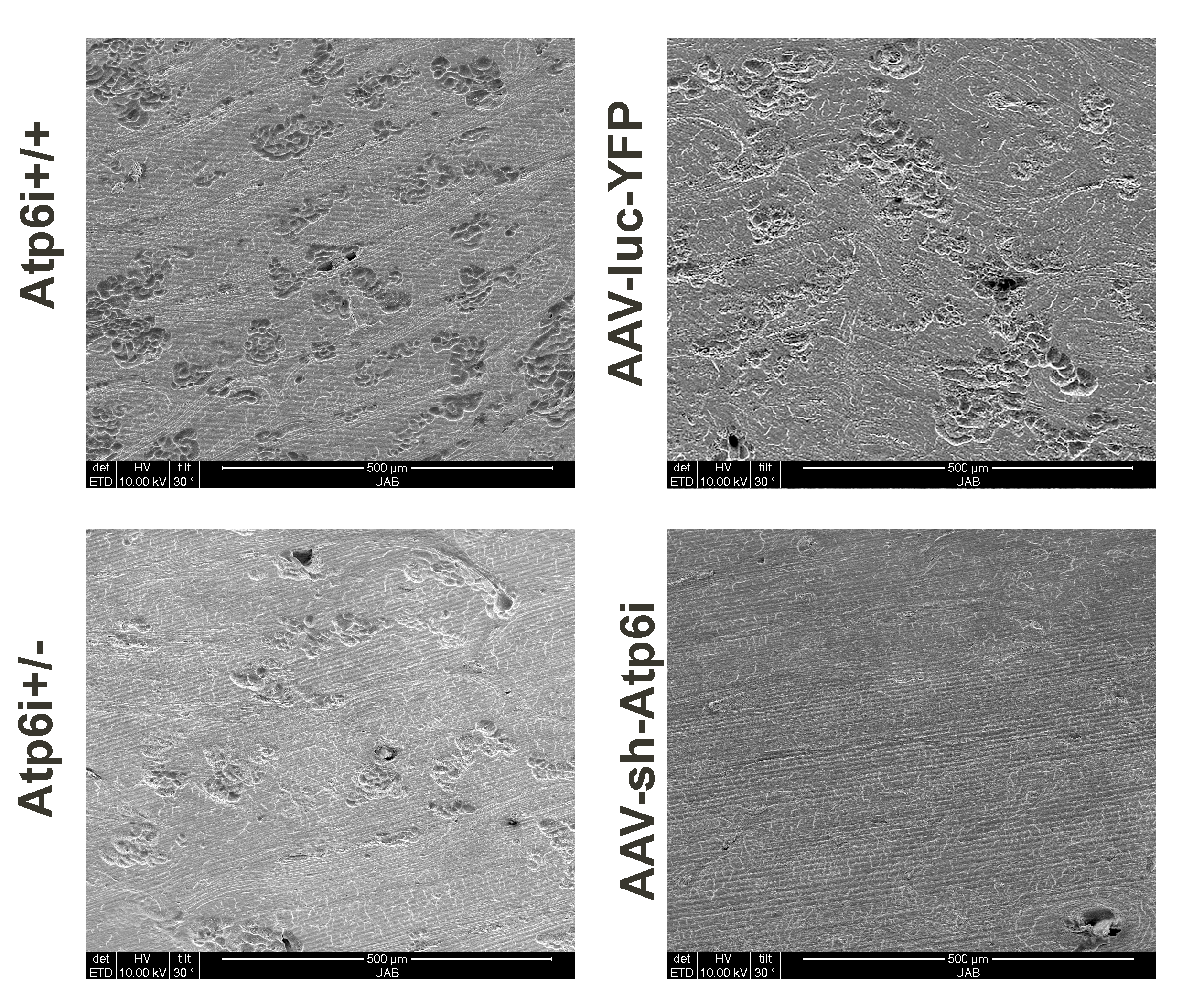

Supplement: S2 File — (TIF) [file pone.0301147.s001.tif]
